# Array-CGH in patients with Kabuki-like phenotype: Identification of two patients with complex rearrangements including 2q37 deletions and no other recurrent aberration

**DOI:** 10.1186/1471-2350-9-27

**Published:** 2008-04-11

**Authors:** Ivon Cuscó, Miguel del Campo, Mireia Vilardell, Eva González, Blanca Gener, Enrique Galán, Laura Toledo, Luis A Pérez-Jurado

**Affiliations:** 1 Unitat de Genètica, Universitat Pompeu Fabra, Barcelona, Spain; 2 CIBER de enfermedades raras (CIBERER), Barcelona, Spain; 3 Programa de Medicina Molecular y Genética, Hospital Vall d'Hebron, Barcelona, Spain; 4 Centre de Regulació Genòmica (CRG), Barcelona, Spain; 5 Clinical Genetics Unit, Hospital de Cruces, Barakaldo, Bizkaia, Spain; 6 Hospital Infanta Cristina, Badajoz, Spain; 7 Hospital Materno Infantil, Unidad de Neurologia Infantil, Las Palmas de Gran Canaria, Spain

## Abstract

**Background:**

Kabuki syndrome (KS) is a multiple congenital anomaly syndrome characterized by specific facial features, mild to moderate mental retardation, postnatal growth delay, skeletal abnormalities, and unusual dermatoglyphic patterns with prominent fingertip pads. A 3.5 Mb duplication at 8p23.1-p22 was once reported as a specific alteration in KS but has not been confirmed in other patients. The molecular basis of KS remains unknown.

**Methods:**

We have studied 16 Spanish patients with a clinical diagnosis of KS or KS-like to search for genomic imbalances using genome-wide array technologies. All putative rearrangements were confirmed by FISH, microsatellite markers and/or MLPA assays, which also determined whether the imbalance was *de novo *or inherited.

**Results:**

No duplication at 8p23.1-p22 was observed in our patients. We detected complex rearrangements involving 2q in two patients with Kabuki-like features: 1) a *de novo *inverted duplication of 11 Mb with a 4.5 Mb terminal deletion, and 2) a *de novo *7.2 Mb-terminal deletion in a patient with an additional *de novo *0.5 Mb interstitial deletion in 16p. Additional copy number variations (CNV), either inherited or reported in normal controls, were identified and interpreted as polymorphic variants. No specific CNV was significantly increased in the KS group.

**Conclusion:**

Our results further confirmed that genomic duplications of 8p23 region are not a common cause of KS and failed to detect other recurrent rearrangement causing this disorder. The detection of two patients with 2q37 deletions suggests that there is a phenotypic overlap between the two conditions, and screening this region in the Kabuki-like patients should be considered.

## Background

Kabuki syndrome (KS), also known as Kabuki make-up syndrome (OMIM 147920), is a multiple congenital anomaly syndrome independently established by Niikawa [1] and Kuroki [2] with an estimated incidence of 1 in 32000 newborns [3]. KS is characterized by specific facial features including long palpebral fissures with eversion of the lateral one-third of the lower eyelid, arched eyebrows with sparseness of the lateral third, a short columella with a depressed nasal tip and prominent ears. This syndrome includes mild to moderate mental retardation, postnatal growth retardation with final short stature, skeletal abnormalities, and unusual dermatoglyphic patterns with prominent fingertip pads. The majority of reported patients have been sporadic with similar sex ratio. Several instances of vertical transmission of KS and KS-like features have been reported, making autosomal dominant inheritance a likely mode of transmission. No consensus diagnostic criteria have been established yet, but based on the clinical findings reported by Niikawa et al [1] five cardinal manifestations were defined to include patients in the KS group (peculiar face, skeletal anomalies, dermatoglyphic abnormalities, mild to moderate mental retardation and postnatal growth deficiency).

Although the cause of KS is currently unknown, numerous cytogenetic abnormalities have been reported in patients with a clinical diagnosis of KS [3]. Matsumoto and Nikawa [4] speculated on a possible microdeletion or microduplication involving contiguous genes as the cause of the disorder, given that the KS patients showed a wide spectrum of multisystemic manifestations and that the majority of patients were sporadic. Milunsky and Huang [5] using classic comparative genomic hybridization (CGH) and fluorescence *in situ *hybridization (FISH) on metaphase chromosomes, found a 3.5 Mb duplication at 8p23.1-p22 in 6 unrelated KS patients suggesting that this alteration may represent a common etiology for the disorder. However, this duplication has not been observed in other multiple patients studied by other groups [6-11] indicating that it is not a common finding in KS.

Maas et al. have also recently reported a *de novo *250 kb deletion disrupting a single gene of unknown function in 20p12.1, *C20orf133*, detected by aCGH in a single KS patient out of 20 [12].

In order to search for putative microrearrangements responsible of KS, we have first screened our 16 patients referred to us with clinical suspicion of KS for 8p22-23 duplications by microsatellite analysis. Subsequently, in 10 patients with adequate DNA, we performed a genome wide survey of DNA aberrations with two different technologies: an in-house printed BAC array, with a global coverage of 23% of the genome and higher density in hot-spot candidate regions (located between segmental duplications and subtelomeric positions) and a commercial oligonucleotide array that spans coding and noncoding sequences with an average spatial resolution of ~100 kb (Agilent G4410B).

## Methods

### Patients and controls

A total of 16 individuals (9 males and 7 females, age range 1–20 years) with clinical manifestations overlapping with the KS phenotype and referred from several Spanish hospitals were included in the study. All referring physicians filled out a basic clinical datasheet and sent pictures of their patients. They all shared at least some of the facial features of KS that lead to the suspicion of this diagnosis along with mental retardation (mild to moderate in 12 cases, severe in 2). Other features included short stature (7/16), skeletal anomalies (13/16), prominent fingertip pads (14/16), and major visceral anomalies or dysfunction including congenital heart defect (6/16), urinary anomalies (3/16), and anogenital anomalies (4/16). We summarized the major clinical findings in Table 1. Two patients, KS13 and KS14, had been previously reported [13].

**Table 1:** Summary of major clinical findings in Kabuki patients.

**Sample**	**KS1**	**KS2**	**KS3**	**KS4**	**KS5**	**KS6**	**KS7**	**KS8**	**KS9**	**KS10**	**KS11**	**KS12**	KS13*	KS14 *	**KS15**	**KS16**	**2q37.3 deletion**
**Age**	20 months	5 years	10 years	10 years	4 years	7 years	2 years	2 years	8 years	8 months	12 years	10 years	20 years	17 years	8 years	8 months	
**Gender**	Female	Female	Male	Female	Male	Female	Male	Male	Male	Male	Male	Male	Female	Male	Male	Female	
**Prenatal growth restriction**	+	-	+	-	-	+	-	-	+	-	-	-	-	-	-	+	-
**Postnatal growth restriction**	+	-	-	-	-	+	+	+	-	-	-	+	-	+	-	+	+
**Microcephaly**	-	-	-	+	-	-	+	-	+	+	-	+	-	-	-	+	+
**Craniofacial abnormality**																	
**Prominent forehead**	-	+	-	-	-	-	-	-	-	-	-	-	-	+	-	-	+
**Long palpebral fissures**	-	-	-	-	+	+	+	+	+	+	-	+	+	+	+	+	-
**Lower palpebral eversion**	-	-	+	-	-	-	+	-	+	-	+	+	+	+	+	+	-
**Arched eyebrows**	-	+	+	+	+	-	+	-	-	-	-	-	+	+	-	+	+
**Deep set eyes**	-	-	-	-	-	-	-	-	-	-	-	-	-	+	-	-	+
**Blue sclerae**	+	+	+	-	+	-	+	-	-	+	-	-	-	-	-	+	-
**Short, upturned nose**	-	-	-	-	-	-	-	-	-	-	-	-	-	-	-	-	+
**Depressed nasal tip**	+	+	+	+	+	+	+	-	+	+	-	+	-	+	-	+	-
**Preauricular pit**	-	-	-	-	-	-	-	-	-	-	-	+	-	-	-	-	-
**Prominent or dysmorphic ears**	+	+	+	-	+	+	+	+	+	-	-	+	+	+	-	+	+
**Cleft palate**	BU	-	-	-	BU	-	-	-	BU	-	-	-	-	-	+	+	-
**Tented upper lip**	-	+	+	+	+	-	+	-	-	+	+	+	+	-	+	-	-
**High-arched palate**	+	-	-	+	-	+	+	+	+	-	-	+	+	+	+		+
**Strabismus**	-	+	-	-	-	-	-	mo	-	-	-	+	+	-	-		+
**Abnormal Dentition**	-	+	-	-	-	-	-	-	-	-	+	-	+	+	-		-
**Skeletal abnormality**																	
**Brachydactyly one finger or general**	+	+	+	+	+	+	+	-	-	-	-	+	+	-	-	+	+
**Short 5th finger with clinodactyly**	-	-	+	+	-	-	-	-	-	-		+	+	-	-	+	-
**Abnormal vertebrae/ribs**	-	RH	-	-	-	-	-	-	-	TR	-	-	+	DR	-		+
**Scoliosis**	-	-	-	-	-	-	-	-	-	-	+, dorsal	-	-	-	-		+
**Joint laxity**	+	-	-	+	-	-	+	-	-	-	-	+	-	-	-		+
**Prominent finger tip pads**	+	+	+	+	+	+	+	+	+	+	-	+	+	+	+		-
**Neurologyc abnormality**																	
**Hypotonia in infancy**	+	+	-	+	+	+	+	+	+	+	-	+	+	+	+	-	+
**Mental retardation**	mild	mild	-	severe	mild	mild	mild	mild	mild	severe	-	mild	mild	mild	mild	mild	+
**Seizure**	+	+	+	-	+	-	+	-	-	-	-	-	-	-	-	-	+
**Visceral abnormality**																	
**Cardiovascular anomaly**		VSD	-	-	-	-	ASD	-	-	PDA	-	VSD, RAA, ASA	-	VSD	-	VSD	+
**Renal and urinary tract anomalies**		-	-	-	-	NB	-	-	-	PD		-	-		-	PD	+
**CNS malformation**	BA, HCC, CV		-	-	-		-	-	-	-	-				-		+
**Anogenital anomaly**		APA	-	-	MG	-	-	-	NP	-	-	-	-		-	HC, APA	+
**Hearing**																	
**Recurrent otitis media**		+	-	+	-	-	+	-	-	-	-	+	-	-	-		+
**Hearing loss**	+	-	-	+		SN		+	-	-	-	-	-	-	-		+
**Other**											TDAH, AS, SD		AS, M	MA, HH			many
**Analyzed by CGH array**	-	+	-	-	-	+	+	+	+	+	-	+	-	+	+	+	

APA = anteriorly place anus; AS = Astigmatism; ASA = aberrant subclavian artery; ASD = Auricular septal defect; BA = brain atrophy; BU = bifid uvula; CV = Cerebellar vermis; DR = Deformed ribs; HCC = hypoplastic corpus callosum; HC = hypertrophic clitoris; HH = hiatal hernia; M = Myopia; MA = metatarsus adductus; MG = Macrogenitalia; NB = neurogenic bladder; NP = narrow penis; PD = pelvis dilatation; PDA = Patent Ductus Arteriosus; RAA = right aortic arch; SD = School delay; SN = sensori neural; TR = thin ribs; VSD = Ventricular septal defect; RH = rib hypoplasia; * Patients previously published [13]

All patients had normal karyotypes (variable banding resolution 400–650) except for KS14 who had a balanced translocation inherited from his mother, 46,XY,t(15;17)(q15;q21) [13]. Patient KS12 had also been evaluated for the 22q11.2 deletion by FISH, with negative result. Blood samples from the patient and parents were obtained under institutional review board-approved informed consent and genomic DNA was extracted by the salting out method using the Puregene^® ^DNA purification Kit (Gentra Systems). Poor DNA quality or scarce amount, unsuitable for some of the studies, was obtained from 6 samples.

DNA samples isolated from peripheral blood from 120 healthy unrelated controls (anonymous blood donors) were used to prepare reference pools (50 males and 50 females) for hybridization experiments. DNAs from 20 control individuals (10 males and 10 females) as well as patients with other disorders (n = 130) were used in order to define pathological changes in DNA dosage, and to estimate and compare frequencies of CNV for association studies.

### Microsatellite analysis

We analyzed four microsatellite markers (D8S503, D8S520, D8S550 and D8S552) distributed along the 8p22-23.1 region, using publicly available primers and standard PCR conditions. To detect and/or discard a putative duplication at those loci, we searched for extra alleles and/or compared microsatellite allele peak ratios of patients with those of their parents and controls.

### Array-CGH

We have designed a microarray of 5442 large insert DNA fragments (BACs) with a global coverage of 23% of the euchromatic genome and much higher density in hot-spot candidate regions, such as those located between segmental duplications and all subtelomeres, named HSBA (Hot-Spot-BAC-Array). BACs were selected from the Sanger 1 Mb array kindly donated by JC Cigudosa, and complemented with clones from the 32 K library [14]. The distribution of BACs in the array is not homogeneous, but the average spacing between consecutive clones is 0.5 Mb and the maximum 1.2 Mb [see additional Table 1]. BAC amplification was done by DOP-PCR as previously described [15] and then spotted in 50% DMSO in triplicate on Corning UltraGaps slides (VersArray ChipWritter Pro™ System, Bio-Rad). Samples of 400 ng of genomic DNA from each of the 10 patients with good quality DNA were labeled by random priming with Cy3-dCTP and Cy5-dCTP and hybridized in dye-swap experiments against a reference pool of the same gender (either 50 females or 50 males). Hybridizations were done as described by Wang et al. [16] using dextrane sulfate and formamide as hybridization solution.

We also used a commercially available 60-mer oligonucleotide microarray for CGH, with probes located in coding and noncoding sequences at an average spatial localization of ~1/35 kb (Agilent G4410B). We hybridized the samples following the manufacturer's protocol (v4), in dye-swap experiments against the same reference pool.

For both arrays, images were obtained with an Agilent Microarray Scanner (G2565BA) (Agilent Inc., Palo Alto, CA) and raw data were extracted with the Genepix Pro 6.0 software (Axon, Inc.) using the irregular spot finding features with default flagging settings. We normalized the log2 ratios between the test sample and the reference pool (M values) by using the print tip loess method. Simple thresholds were used in order to detect DNA imbalances. HSBA clones with all M replicate values outside the arbitrary range of |0.2| in both hybridization experiments were considered as amplified (>0.2) or deleted (<-0.2). Since oligonucleotide based arrays are noisier than BAC arrays due to the smaller size of the probes and the paucity of internal replicates [17], more stringent analyses were done with G4410B data to minimize false positive results. A potential rearrangement was considered only when 3 out of 3–5 consecutive probes gave M values outside |0.3|, then obtaining an average resolution of ~1/100 kb [18]. The arbitrary thresholds were chosen in both cases based on our previous lab experience (Vilardell et al. submitted article "Statistical assessment of sources of systematic variation in aCGH experiments").

### Validation experiments

Confirmation of the CGH array results was done with different methodologies such as FISH on interphase and metaphase cultured lymphocytes, and/or microsatellite markers segregation and multiple ligation-probe amplification (MLPA) [19] on genomic DNA.

#### FISH

BAC clone DNA was extracted by conventional alkaline lysis from glycerol stocks or by DOP-PCR amplification. The obtained DNA was fluorescently labeled using Spectrum-Red-dUTP (Vysis/Abbott) by nick translation and hybridized by conventional methods on metaphase chromosomes prepared from cultured lymphocytes from patient samples.

#### MLPA

A total of 100 ng of genomic DNA from each sample was subject to MLPA using specific synthetic probes [see Table 2 in additional file 1] designed to target the specific CNV detected by aCGH. The MLPA reactions were analyzed on an ABI PRISM 3100 Genetic analyzer according to manufacturers' instructions. Each MLPA signal was normalized and compared to the corresponding peak height obtained in at least 5 control DNA samples.

### DNA paternity testing

Paternity was tested in patients with *de novo *rearrangements KS2 and KS14. From each sample of the trios (father, mother, child) we analyzed four microsatellite markers of chromosome 7 (D7S642, D7S636, D7S2426, D7S688) and two microsatellite markers of chromosome 15 (D15S1014, D15S207) using publicly available primers and standard PCR conditions.

### Statistical methods

Statistical analysis was performed using the R-platform. Multi test effect was corrected by the Benjamini-Hochberg (BH) method [20] when it was necessary. In all cases statistical significance was considered for corrected p values < 0.05. To search for putative association of CNVs with KS, we compared the absolute frequencies of CNVs (number of deletions, amplifications and no changes for each CNV) between KS samples and controls. Differences were assessed with the chi-square test using resampling with a 10,000 sample size instead of the reference chi-square distribution in order to avoid problems with the reduced sample size. Furthermore, Fisher exact test was applied when we compared the number of times that changes and no-changes between Kabuki and controls were detected. We repeated all tests twice, first with entire sample of patients, and then excluding the two cases with known causal rearrangements.

## Results

### Lack of duplication at 8p22-23.1

Four microsatellite markers covering the 8p22-23 region previously reported as duplicated were genotyped in all patients using parental samples for allelic identification and comparative sizing. We also analyzed 20 control individuals to establish the normal relative peak ratios in different allelic combinations. All patients were informative (heterozyogous) at two or more of the analyzed loci and none of the markers showed evidence of duplication in this region (data not shown).

### Detection of genomic imbalances in KS samples by aCGH

Using the HSBA array in the 10 KS samples with available good quality DNA, a total of 109 autosomic BAC clones detected copy number alterations (41 gains, 47 losses and 21 BACs with both gains and losses). Sixty six of these BACs had previously been reported by different authors as located within polymorphic CNVs [21-29] and 19 were also detected as common variants in our control population (20 controls and 130 patients with unrelated disorders) [see Table 3 in additional file 1]. The remaining 24 BACs corresponding to genomic imbalances in 7 regions were present only in KS samples and were consequently considered as candidate regions for pathogenic involvement in our patients (Table 2). Using the oligonucleotide G4410B array, we validated most results of the HSBA array and obtained additional information for regions that were not covered by the BAC array (Table 2).

**Table 2:** Patient specific rearrangements detected by aCGH

**Sample**	**Chr**	**Band**	**Start**	**End**	**Alteration**	**Confirmed**	**Origin**	**Genes (within the BAC clone)**	**Genes (Agilent Probe)**
**KS2**	2	2q36.3-2q37.3	226400869 (225995608)	237338113	Gain	1,2	*de novo *(Maternal)	*COL4A4, COL4A3, SP140, LOC93349, SP100, INPPSD, ATG1GL1, SAG, DGKD, USP40, UGT1A8, UGT1A10, UGT1A9, UGT1A7, UGT1A6, DKFZp762E1312, TRPM8, SH3BP4 CENTG2 IQCA, CHKOR1*	
	2	2q37.3	237607249	242166437 (telomere)	Loss	1,2	*de novo *(Maternal)	*COPS8, UBE2F, SCLY, LOC339768 TRAF31P1, ASB1 NDUFA10, ORGB2, ORGB3, MYEOV2, OTOS SEPT2, FARP2, STK25*	
**KS6**	14	14q23.1	57693088 (57541570)	58084505 (58177255)	GAIN	3,4	Paternally inherited		*ACTR10, PSMA3, ARID4A, UNQ9438, TIMM9, KIAA0586*
				54849232			Maternally		
**KS7**	5	5q11.2	54497449 (54489157)	(54849173)	GAIN	3,4	inherited	*FLJ37927, UNG2, DHX29, SKIV2L2*	*UNG2; SKIV2L2; PPAP2A*
**KS9**	17*	17q12	30619177 (30610975)	30792737 (30797213)	LOSS	3,4	Maternally inherited	*FLJ34922*	*SLFN11, SLFN12*
	2	2q37.2-2q37.3	235511580 (235053304)	242166437 (telomere)	Loss	1,2,3	*de novo *(Paternal)	*SH3BP4 CENTG2 IQCA, CHKOR1, COPS8, UBE2F, SCLY, LOC339768 TRAF31P1, ASB1 NDUFA10, ORGB2, ORGB3, MYEOV2, OTOS SEPT2, FARP2, STK25*	
**KS14**	16*	16p11.2	29546566 (29532360)	30106101 (30271412)	Loss	2,3,4	de novo (Maternal)	*BOLA2, SPN, C16orf54, KIF22, MAZ TAOK2, HIRIP3, FLJ90652, DOC2A, DKFZP434I2117, ALDOA, PPP4C*	*SPN, QPRT, C16orf54, KIF22, MAZ, TAOK2, HIRIP3, FLJ90652, DOC2A, DKFZP434I2117, ALDOA, PPP4C, TBX6, YPEL3, GDPD3, MAPK3, CORO1A*

1 = FISH, 2 = Microsatellite markers, 3 = Agilent oligo array G4410B, 4 = MLPA

Note that in the "Start and End" positions we have included the neighboring positions that were not altered in parentheses

### Confirmation and characterization of the genomic imbalances

**Figure 1:**
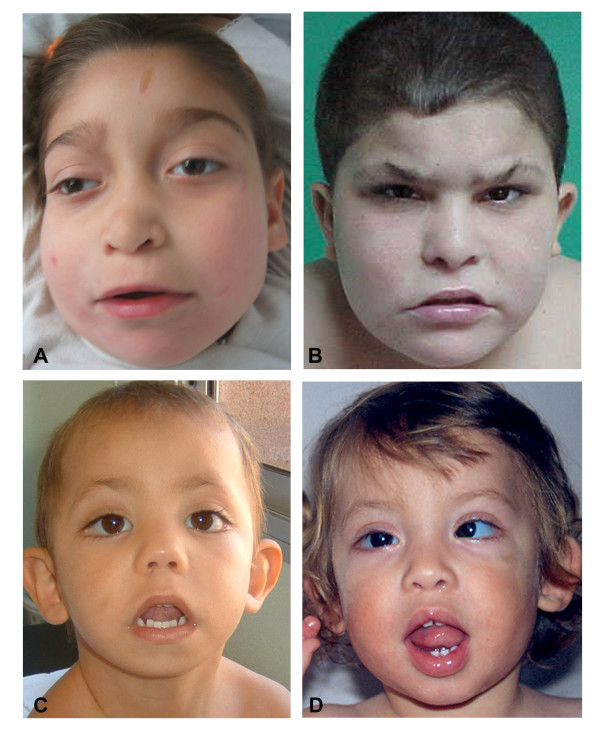
**Facial phenotype of 2 Kabuki-like (A:KS2; B:KS14) and 2 Kabuki patients (C:KS7; D:KS12). **Note that KS2 and KS14 also show some features characteristic of the 2q37 deletion.

**Figure 2:**
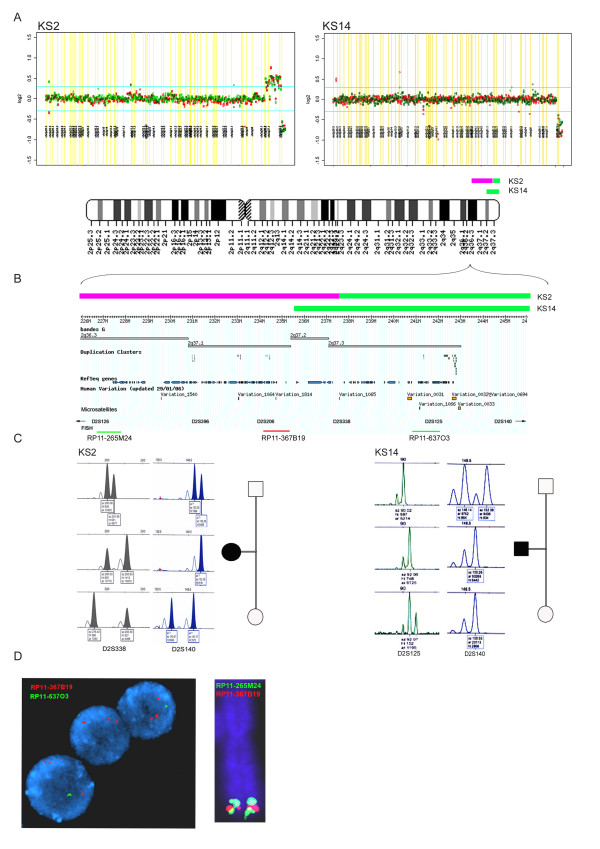
**Detection and validation of the 2q genomic imbalances.** A and B: Plot of M-Values of clones on chromosome 2 and ideogram showing alterations in KS2 (A) and KS14 (B) patients. C: Microsatellite analysis showing the parental origin of these *de novo *alterations (KS2: Maternal chromosome; KS14: Paternal chromosome). D: FISH analysis with BACs RP11-367B19 (red) (2q37.2), RP11-637O3 (green) (2q37.3) and RP11-265M24 (green) (2q36.3) probes confirming *de novo *aberration originated in the maternal chromosome.

The patient specific rearrangements found were the following (Table 2):

**KS2 **(Figure 1): The HSBA showed a duplication of 22 clones of chromosome 2q36.3-2q37.3 and a deletion of 7 clones in 2q37.3-qter. Microsatellite analysis with markers D2S126, D2S396, D2S206, D2S338, D2S125 and D2S140, as well as FISH studies with BACs RP11-367B19 at 2q37.2 (red), RP11-637O3 at 2q37.3 (green) and RP11-265M24 at 2q36.3 (green) confirmed a *de novo *aberration originated in the maternal chromosome consisting in a ~11 Mb inverted duplication in 2q36.3 and a contiguous ~4.5 Mb terminal deletion of 2q37-qter previously unrecognized in the G-banded karyotype (Figure 2). Paternity was confirmed using 6 additional microsatellite markers.

**Figure 3:**
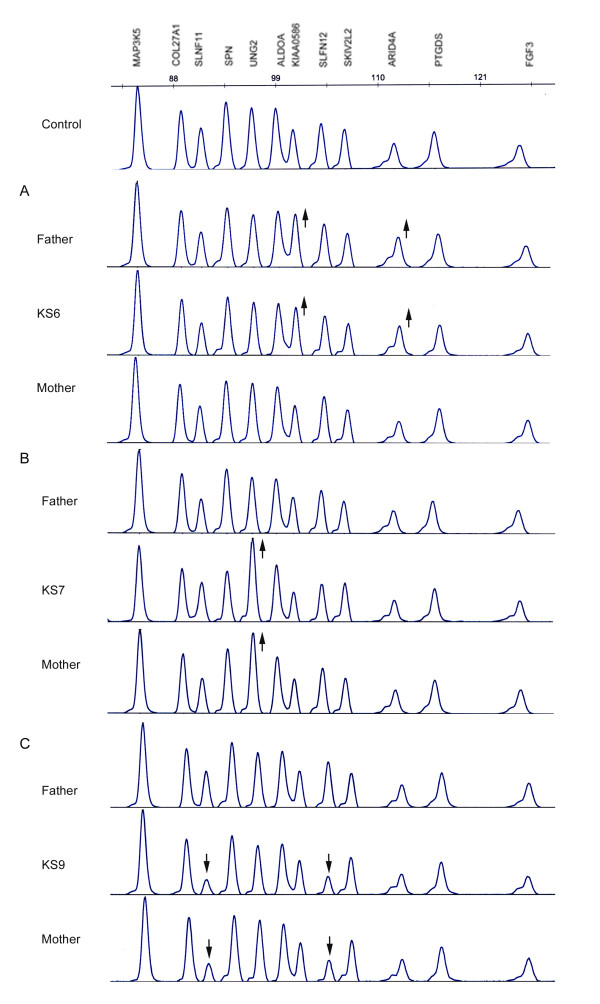
**Electropherograms of MLPA assay with specific synthetic probes.** A: Duplication pattern of probes for the *ARID4A *and *KIAA0586 *genes in the KS6 patient and her father. B: Duplication pattern of the *UNG2 *probe in KS7 patient and in his mother. C: Deletion pattern of two specific probes for *SLFN11 *and *SLFN12 *genes in the KS9 patient and his mother.

**KS6: **No specific alterations were found with HSBA but the G4410B array revealed an amplification of 12 consecutive probes encompassing ~392 Kb in 14q23.1. An MLPA assay with two specific probes (*ARID4A *and *KIAA0586 *genes) showed that this duplication was a paternally inherited CNV (Figure 3).

**KS7: **A duplication of one BAC clone (RP11-506H20) located in 5q11.2 was identified by the HSBA and then validated by nine probes of the same region in the G4410B array, defining a duplication of ~285 Kb. An MLPA assay with a probe located in the *UNG2 *gene showed a duplication pattern in the patient and his mother, indicating that this duplication was also an inherited CNV (Figure 3).

**KS9: **The HSBA array showed a deletion of one clone in chr17q12, ratified by a deletion of three consecutive probes of the Agilent array encompassing ~173 Kb in the same interval containing the *SLFN11 *and *SLFN12 *genes. MLPA with two specific probes for *SLFN11 *and *SLFN12 *genes showed a deletion in the patient and his mother also indicating an inherited CNV (Figure 3).

**KS12**: The HSBA array showed the deletion of one BAC at chr11q14.3 that was not confirmed by the G4410B array. Using the defined criteria, five additional chromosomal regions were identified by the oligo G4410B array. However, specific MLPA probes were designed for the genes present in those regions and none of the presumed alterations were confirmed despite the coincidence of some MLPA and G4410B probes.

**Figure 4:**
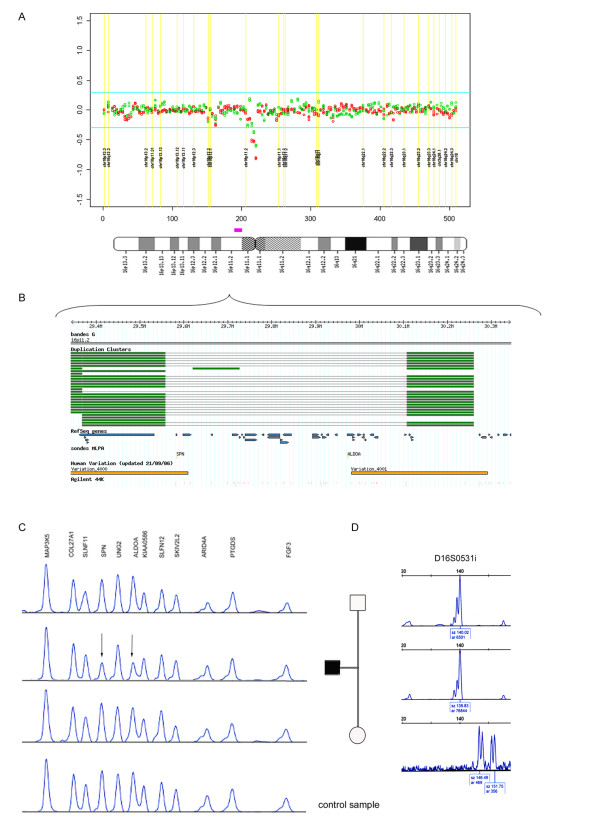
**Detection and validation of 16p11.2 deletion in KS14.** A and B: Plot of M-Values of clones on chromosome 16 and ideogram showing the deleted interval in the patient. C: Deletion patterns of MLPA at specific 16p11.2 probes (*SPN *and *ALDOA *genes) showing the *de novo *event in the patient with respect to parental samples and a control. D: Microsatellite analysis showing the maternal origin of the *de novo *deletion

**KS14 **(Figure 1): A previous karyotype of this child and his mother had showed an apparently balanced translocation 46,XY,t(15;17)(q15;q21) and no other rearrangement [13]. The HSBA revealed a deletion of 10 terminal BAC clones on chromosome 2q37.2-2qter and an additional deletion of 3 BACs at chr16p11.2 (Figure 4). The G4410B Agilent array confirmed a deletion of 101 probes covering ~7.2 Mb of 2q37.2-ter, as well as the deletion of 33 probes covering ~0.5 Mb at 16p11.2. Microsatellite analysis at loci D2S206, D2S338, D2S125 and D2S140, confirmed a *de novo *2q37 deletion in the paternally inherited chromosome, while the microsatellite D16S0531i revealed a *de novo *16p11 deletion of maternal origin (Figure 2). MLPA with probes for two different genes (*SPN *and *ALDOA*) further confirmed that the 16p11.2 deletion was also a *de novo *event (Figure 4). Paternity was confirmed using 6 additional microsatellite markers.

We did not find specific genomic imbalances in any of the remaining four KS samples analyzed by both aCGH technologies (KS8, KS10, KS15 and KS16).

### Association analysis with CNVs

We then designed an MLPA panel including probes for all rearrangements only detected in our patients and used to screen all samples, including those that had not been studied by aCGH (KS1, KS3, KS4, KS5, KS11 and KS13). None of the rearrangements was detected in any other sample.

In order to detect possible associations of CNVs with KS, we performed adjusted chi-square tests comparing the frequencies of the 85 CNV regions detected by our analyses [see Tables 3 and 4 in additional file 1] in KS samples versus controls. We included as controls both, ours and those previously published by other control groups, mainly HapMap samples [30]. We compared the presence or absence of any of the alterations, as well as the potential differences based on the type of alteration (amp/del/none). Excluding the chromosome 2 rearrangements, we detected 13 CNVs on average in KS samples and 12.4 in our control population (no significant difference). Using the chi-square and Fisher's test we did not detect associations with any CNV or CNV combination (corrected and raw p values > 0.05) in the KS sample group.

## Discussion

KS is a developmental syndrome with multisystemic involvement but variable expressivity, most cases being sporadic with a few exceptions of possible vertical transmission in an autosomal dominant pattern. This presentation highly suggests that KS could be caused by a recurrent genomic disorder affecting more than one gene [4]. To test this hypothesis, we have studied 16 Spanish patients with clinical features suggestive of a diagnosis of KS or KS-like, using molecular tools aimed at detecting cryptic genomic imbalances. We decided not to use strict clinical inclusion criteria with the idea that the molecular characterization of patients with overlapping phenotypes may provide clues to the final identification of the molecular basis of specific syndromes.

We first ruled out in all cases gene dosage alterations at 8p22-8p23.1, the region previously reported to be duplicated in six American KS patients (four Caucasian, one African-American, and one Haitian) [5]. Our results, as recently reported by others [6,8-11,31,32], do not support the claim that genomic rearrangements of 8p22-8p23.1 are a common cause of KS.

To screen for putative submicroscopic genomic copy number alterations in our KS and KS-like population, we used two high throughput technologies based on aCGH with different genomic coverage, a BAC array with high density in putative hotspots for genomic disorders and a gene-based oligo array. We did not detect any copy number changes in the 20p12.1 region recently associated with KS, covered by two BACs in the HSBA and 15 probes in the G4410B array, further indicating that rearrangements at this locus are not a common cause of KS [12]. Surprisingly, we found rearrangements of terminal 2q in two patients (KS2 and KS14) with clinical manifestations overlapping with the KS phenotype. In patient KS2, we detected an inverted duplication with terminal 2q deletion (invdup-del2q, invdup 11 Mb-del 4.5 Mb). In patient KS14, who had a karyotype with an apparently balanced translocation (t(15,17)) inherited from his mother [13], we found two *de novo *unbalanced rearrangements: a 7.2 Mb terminal deletion of 2q37 in the paternal chromosome and a ~0.5 Mb interstitial deletion of 16p11.2 in the maternal chromosome. Patterns of complex rearrangements affecting several chromosomes and mostly generated in the paternal meiosis have been previously described by aCGH in some patients with apparently balanced *de novo *translocations [33,34], suggesting a common mechanism for those rearrangements. However in KS14, no alterations were detected with any of the arrays in the proximity to the breakpoints of the cytogenetically balanced translocation inherited from the mother.

Therefore, we have detected overlapping rearrangements of the long arm of chromosome 2 in two KS-like patients. Although the rearrangements appear complex involving dosage imbalance for additional chromosomal regions in both cases, the region of overlap consists of a terminal 2q37 deletion of ~4,5 Mb. The long arm of chromosome 2 has a specific structure related to the ancestral fusion site of two primate subtelomeric regions at 2q13 and a subtelomeric duplicated region of DNA enriched with CpG islands [35]. To date, more than 60 patients with 2q37 deletions and a few rare cases with inverted duplications of 2q have been described. In fact, inverted duplications are thought to be recurrent rearrangements always associated with distal deletions [36]. Almost all patients with 2q37 deletions have some degree of mental retardation, some with autistic features, and facial dysmorphism. Brachymetaphalangism has been reported in approximately of 50% of patients while congenital heart defects, predominantly atrial or ventricular septal defects or more complex defects, are found in 20% of patients with 2q37 monosomy [37]. Phenotype-genotype correlations have defined candidate regions to harbor the genes responsible for congenital heart defects, brachymetaphalangysm and neurobehavioural problems [37]. However, considerably clinical variability was apparent even for patients with similar breakpoints pointing to the existence of modifiers, epigenetic or environmental phenomena also involved in phenotypic variability.

Even though their specific common facial dysmorphism (a round face with flat nasal bridge, dysmorphic ears often prominent, deep-set eyes, anteverted nares and thin upper lip) do not include long palpebral fissures with lateral eversion of the lower third, bulbous depressed nasal tip, or prominent ears with ear pits, characteristic of KS, the private features of these two patients (Figure 1) made the diagnosis of KS a possibility for our clinicians, considering brachymetaphalangy with prominent fingertip pads, a depressed nasal tip and prominent ears, mild to moderate mental retardation and heart defects were present in both. The phenotype in both patients was likely caused by additive effects of gene dosage imbalance of the 2q37 deletion that included the critical interval for most phenotypic features, and the additional rearrangement (2q36 duplication in KS2 and 16p11.2 deletion in KS14). However, given this phenotypic overlap, we believe that the diagnosis of a 2q37 deletion should be considered in patients with KS-like phenotypes where the characteristic eyes are less evident, a feature that is often obvious only after 3 years of age.

The 16p11.2 interstitial deletion additionally found in KS14 corresponds to a region flanked by segmental duplications that has been reported recently as one of the most common recurrent genomic imbalances associated with autism and developmental delay [38-40]. Interestingly, both *de novo *and inherited deletions and duplications of the region have been reported in ~1% of the ASD cases, indicating that the 16p11 region carries a substantial susceptibility risk for autism. The same region was found included in a new microdeletion syndrome in 16p11.2-p12.2 reported in patients with mental retardation, dysmorphic face and eye anomalies, even though the 0.5 Mb deletion of our patient is not part of the common interval but is included in the largest deletion reported in just one patient [41]. However, KS14 did not show autistic features nor eye anomalies, even though also several 2q37.3 deletion syndrome patients have been reported to have autistic features.

This region harbors multiple genes that could be dosage sensitive and involved in the regulation of developmental processes, one example being *TBX6*. Mouse embryos with reduced levels of Tbx6 show defective somite patterning of the paraxial mesoderm [42]. Therefore, it is tempting to speculate that some of the phenotypic features of KS14, such as the abnormal ribs, could be related with additive effects involving haploinsufficiency for *TBX6*. The finding of different parental origins for the chromosome 2q27 and the chromosome 16p11.2 deletions suggests that the rearrangements are not mechanistically related and their association must have occurred by chance.

The array technologies allow a rapid and automated evaluation of CNVs with genome-wide resolution.

We have detected three additional genomic alterations in KS patients not found in other samples and not reported as polymorphic CNVs in the published studies [21-27]. However, all three were inherited from a phenotypically normal progenitor in each case, suggesting that they are most likely polymorphic and non pathogenic rare CNVs. Given that most CNVs are quite rare in the population, an alternative hypothesis to the classic genomic disorder that could be the cause of disease, KS in this case, would be the additive effects of two or more rare CNVs. In our patient population, we detected the presence of a total of 85 CNVs, 66 of which were previously reported as polymorphic [30] and the other 19 were identified as new variants in our control populations. No patient specific combination of CNVs was found in the patients and the frequencies of the 85 detected CNVs between KS and controls showed no significant (p > 0.05) associations, suggesting those regions do not contribute to the KS phenotype.

## Conclusion

A major conclusion of our study is that we were unable to find any common rearrangement causally related to KS. The combination of a high resolution BAC array specifically enriched for putative hotspot regions and a commercial 44 k oligo array (average resolution of 100 kb) failed to identify any common deletion or duplication in typical KS and KS-like patients, as previous reports using 1 Mb resolution BAC arrays. The inclusion of two patients in which a terminal 2q37 deletion syndrome could have been clinically suspected, suggests there is some phenotypic overlap among the two conditions, and this diagnosis should be kept in mind when evaluating patients with KS-like phenotypes. This study also supports that the 3.5 Mb duplication at 8p23.1-p22 is not the principal cause nor a common cause of KS [6,8-11,31,32].

Although limited by the resolution of our arrays, our results indicate that a recurrent copy number genomic rearrangement affecting contiguous genes is unlikely to be the common cause of KS. Other type of structural changes, such as cryptic inversions, can not be ruled out with the applied technology and remain a possible explanation. Alternatively, KS could be caused by *de novo *genetic or epigenetic dominant alterations affecting a gene with pleiotropic effects, given that both concordant and discordant monozygotic twins have been reported [43,44]. Further studies with higher resolution array platforms are indicated for typical and atypical patients suspected of having KS, since no strict diagnostic criteria have yet been established and no common etiology has yet been found.

## Competing interests

The author(s) declare that they have no competing interests.

## List of abbreviations

KS, Kabuki syndrome; CNV, copy number variations; aCGH, comparative genomic hybridization array; FISH fluorescence *in situ *hybridization; HSBA (Hot-Spot-BAC-Array); MLPA, multiple ligation-probe amplification; BH, Benjamini-Hochberg method.

## Authors' contributions

IC carried out the molecular genetic studies, participated in the design of the study and in the statistical analysis and drafted the manuscript. MdC participated in the collection of all clinical diagnostic data and helped to draft the manuscript. MV performed the statistical analysis. EG performed the array-CGH analysis. BG, EG and LT carried out the clinical evaluation of the patients. LAP conceived of the study, and participated in the design and coordination and helped to draft the manuscript. All authors read and approved the final version of the manuscript.

## Pre-publication history

The pre-publication history for this paper can be accessed here:



## Supplementary Material

Additional file 1 Distribution of BACs in the array HSBA (Hot-Spot-BAC-Array). The data provided includes all the BACs analyzed in the array HSBA. Click here for file
